# Lineage-Specific Patterns of Genome Deterioration in Obligate Symbionts of Sharpshooter Leafhoppers

**DOI:** 10.1093/gbe/evv159

**Published:** 2015-08-10

**Authors:** Gordon M. Bennett, John P. McCutcheon, Bradon R. McDonald, Nancy A. Moran

**Affiliations:** ^1^Department of Integrative Biology, University of Texas, Austin; ^2^Department of Plant and Environmental Protection Sciences, University of Hawaii, Manoa; ^3^Division of Biological Sciences, University of Montana, Missoula; ^4^Department of Bacteriology, University of Wisconsin, Madison; ^5^Institute for Cellular and Molecular Biology, University of Texas, Austin

**Keywords:** *Baumannia cicadellinicola*, methionine, GC content, selection, substitution rates, DNA repair

## Abstract

Plant sap-feeding insects (Hemiptera) rely on obligate bacterial symbionts that provision nutrients. Some of these symbionts are ancient and have evolved tiny genomes, whereas others are younger and retain larger, dynamic genomes. *Baumannia cicadellinicola*, an obligate symbiont of sharpshooter leafhoppers, is derived from a relatively recent symbiont replacement. To better understand evolutionary decay of genomes, we compared *Baumannia* from three host species. A newly sequenced genome for *Baumannia* from the green sharpshooter (B-GSS) was compared with genomes of *Baumannia* from the blue-green sharpshooter (B-BGSS, 759 kilobases [kb]) and from the glassy-winged sharpshooter (B-GWSS, 680 kb). B-GSS has the smallest *Baumannia* genome sequenced to date (633 kb), with only three unique genes, all involved in membrane function. It has lost nearly all pathways involved in vitamin and cofactor synthesis, as well as amino acid biosynthetic pathways that are redundant with pathways of the host or the symbiotic partner, *Sulcia muelleri*. The entire biosynthetic pathway for methionine is eliminated, suggesting that methionine has become a dietary requirement for hosts. B-GSS and B-BGSS share 33 genes involved in bacterial functions (e.g., cell division, membrane synthesis, metabolite transport, etc.) that are lost from the more distantly related B-GWSS and most other tiny genome symbionts. Finally, pairwise divergence estimates indicate that B-GSS has experienced a lineage-specific increase in substitution rates. This increase correlates with accelerated protein-level changes and widespread gene loss. Thus, the mode and tempo of genome reduction vary widely among symbiont lineages and result in wide variation in metabolic capabilities across hosts.

## Introduction

Many insects with specialized diets rely on microbial symbionts to supply unavailable nutrients. Dramatic genome degeneration is inevitable in these symbionts as they converge upon a minimal gene set for nutrition and basic, albeit incomplete, cellular functions (McCutcheon and Moran 2012). However, gene loss can be uneven, and lineages within a single symbiont clade can differ by tens to hundreds of genes (Moran and Bennett 2014). The losses of metabolic capabilities in some symbionts are associated with their host’s acquisition of additional symbiotic partners or of novel trophic niches (van Ham et al. 1997; Sabree et al. 2012; Husnik et al. 2013; Sloan and Moran 2013). Other differences among symbiont genomes are presumably the result of reduced selection, increased mutation rates, and stochastic processes (Moran 1996; Sabree et al. 2010; Rio et al. 2012; Bennett et al. 2014; Dietz et al. 2015; Gottlieb et al. 2015; Wernegreen 2015; Williams and Wernegreen 2015). For lineages within a symbiont clade, it remains unclear to what extent gene sets are conserved due to shared functional constraint and to what extent they reflect ecological differences among hosts.

**Fig. 1.—:**
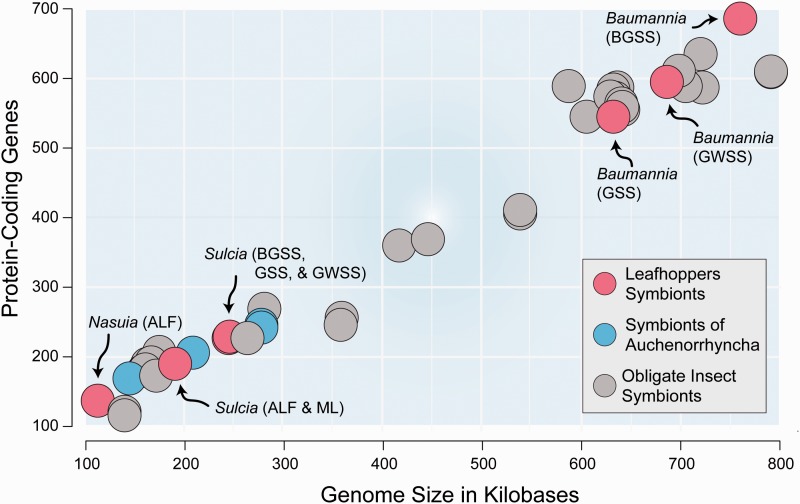
Genome size and protein coding content of the obligate bacterial symbionts in insects with genomes below one megabase. Leafhopper symbionts and other Auchenorrhyncha are color-coded (see inset legend). See text for references.

Sap-feeding insects in the Auchenorrhyncha (Hemiptera: Suborder) harbor a diversity of bacterial symbionts that are responsible for making the ten essential amino acids (EAA) that are generally rare in plant sap and that animals cannot synthesize de novo (see McCutcheon and Moran 2010). These symbiont lineages possess both the tiniest and some of the largest genomes of insect obligate symbionts (fig. 1: Wu et al. 2006; McCutcheon and Moran 2007; McCutcheon et al. 2009; Bennett and Moran 2013; Koga and Moran 2014; Van Leuven et al. 2014). In the leafhoppers (Cicadellidae), one large group within Auchenorrhyncha, hosts typically harbor two symbionts that have partitioned EAA synthesis. Generally, the oldest associate, *Sulcia muelleri* (Bacteroidetes), synthesizes eight EAAs, while a diversity of coresident symbionts are responsible for the remaining two, methionine and histidine (McCutcheon and Moran 2007, 2010; Bennett and Moran 2013; Chang et al. 2015). One of the largest genomes of an insect obligate symbiont sequenced to date belongs to *Baumannia cicadellinicola* (*Gammaproteobacteria*) (fig. 1), which replaced *Nasuia deltocephalinicola* (Betaproteobacteria) 80–175 Ma in xylem-feeding sharpshooter leafhoppers (Cicadellinae: Moran et al. 2003; Takiya et al. 2006). *Baumannia* lineages variably encode pathways for considerable cellular autonomy and redundant metabolisms with their symbiotic partners (Wu et al. 2006; Bennett et al. 2014). Thus, *Baumannia* offers the opportunity to investigate lineage-specific patterns of genome degradation over millions of years of evolution.

**Fig. 2.—:**
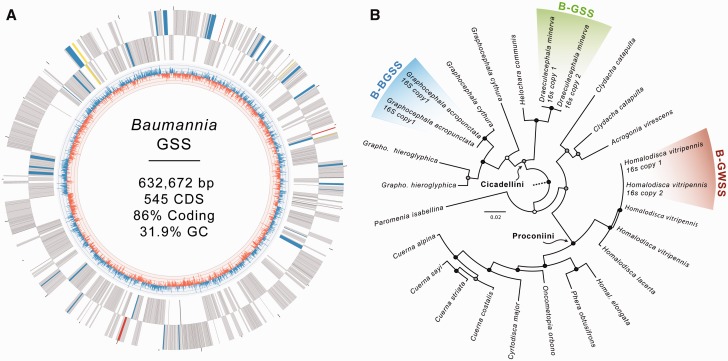
Genome and phylogenetic placement of B-GSS strain. (*A*) Genome plot of genes encoded by B-GSS. The outer two rings show genes encoded on the forward and reverse strand, respectively. The inner graph shows genome-wide GC skew. Genes are color-coded according to the *Baumannia* genomes that share them: gray = core shared; blue = B-GSS and B-BGSS; yellow = B-GSS and B-GWSS; and red = B-GSS only. (*B*) Phylogenetic placement of *Baumannia* strains inferred from 16S rRNA (adapted from Bennett et al. 2014). Strains for which sequenced genomes exist are labeled. Bootstrap support values are indicated by nodal dots (black ≥ 75, gray = 50–74, and white = no support). Outgroups have been trimmed.

To better understand how symbiont lineages diverge as their genomes lose genes, we sequenced *Baumannia* from the green sharpshooter (GSS; *Draeculacephala minerva*), for which the coresident *Sulcia* genome was previously sequenced (Woyke et al. 2010). GSS feeds on xylem of grasses and is a viticulture pest in the Southwestern United States and Hawaii. *Baumannia*-GSS (B-GSS) was selected for its relatively close phylogenetic relationship to *Graphocephala atropunctata* (B-BGSS), which has the largest known *Baumannia* genome (figs. 1 and 2*B*: Takiya et al. 2006; Bennett et al. 2014). Both are placed in the Cicadellini tribe. The more distantly related *Baumannia* of *Homalodisca vitripennis* (B-GWSS; Proconiini) was used to root genomic comparison between B-GSS and B-BGSS.

## Genome Features of B-GSS

B-GSS contains a circular 632,672 bp chromosome (figs. 1 and 2*A*) that is over 50 kb smaller than the other two sequenced *Baumannia* genomes (fig. 1). Except for gene deletions, all three genomes are perfectly syntenic. B-GSS encodes 545 predicted protein-coding genes, 47 tRNAs, two rRNA operons, an ssrA, and seven predicted pseudogenes. B-GSS also contains a 3.5 kb plasmid (pB-GSS), encoding five genes involved in replication, heat-shock, and phospholipase and protease-like functions. All *Baumannia* lineages harbor a similar plasmid. The pB-GSS is similar in protein coding content to the one reported from B-BGSS; however, pB-BGSS is nearly twice the size (6.5 kb) due to gene duplications of *repA* and *ibpA* (Bennett et al. 2014). The B-GSS genome and plasmid are available on GenBank under accessions CP011787–CP011788.

## Lineage-Specific Gene Losses

Genome reduction in the different *Baumannia* lineages reveals lineage-specific patterns of gene loss. The more highly reduced B-GSS genome does not contain a simple subset of the genes found in the two previously sequenced, larger *Baumannia* genomes (fig. 2*A*). Within this set, B-GSS retains only three unique genes, all involved in membrane structure and transport (*ompC* and inner membrane proteins). B-GSS and the more distantly related B-GWSS uniquely share only five genes, involved in translational machinery (*epmAB* and *hflX*) and cellular membrane production (*yidD* and *lpp*). In contrast, B-GSS and the more closely related B*-*BGSS uniquely share 33 genes. These involve bacterial functions that are generally lost from the smallest genomes (Moran and Bennett 2014), including DNA replication initiation *(dnaA*), cell division (*ftsABINQWX*), cell envelope synthesis (*plsX* and *mrcB*), cell growth (*ratA*), and metabolite transport (*gltP* and *mrcB*), among other functions (e.g., complete ubiquinone synthesis). The shared retention by B-GSS and B-BGSS of capabilities for independent cellular function suggests some level of constraint on the loss of certain genetic capabilities from the symbiont genome within the Cicadellini host lineage. Possibly *Baumannia* strains in this host clade are under selection to maintain certain capabilities; the host may be unable to compensate for the loss of these functions (Zientz et al. 2001; Bennett and Moran 2015).

The unique gene losses in the B-GSS genome further indicate that it is converging on the essential functions found in other coprimary symbionts that have far smaller genomes. B-GSS has lost 89 genes that span a broad range of processes, including translation (*tgt*), membrane and transport (*tamAB, cysW, *and *yciC*), cell division (*tig* and *ftsEX*), stringent response (*relA*), and DNA repair (*ung* and *mutM*). Notably, roughly half of all genes lost are involved in pathways and peripheral metabolisms related to vitamin and amino acid synthesis and transport. B-GSS has lost the pathways for B vitamins (biotin, folic acid, pentothenate, and thiamine) and other cofactors (heme). It has further been stripped of amino acid synthesis pathways that are redundant with those of its symbiotic partners (e.g., phenylalanine, cysteine, and lysine; Woyke et al. 2010). This suggests that, while the synthesis of additional vitamins and cofactors might be beneficial for hosts using a xylem diet (Wu et al. 2006), it does not appear to be essential in the long-term. Instead the process of genome decay in auchenorrhynchan symbionts appears to move generally toward limited gene sets involved in central informational processes and synthesizing essential nutrients and little else.

## Unique Loss of EAA Biosynthesis

**Fig. 3.—:**
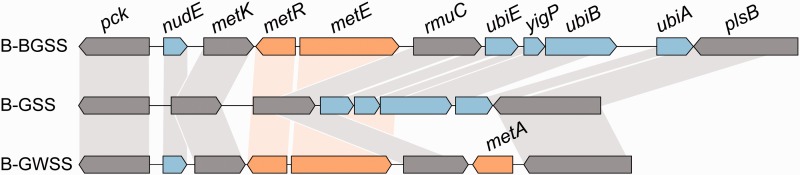
Orthologous gene retention and losses between *Baumannia* strains, highlighting the methionine synthesis. Genes in gray are shared between all three genomes, whereas genes in blue are shared by at least two strains (see connecting lines). Genes that are shaded orange are involved in methionine synthesis.

Remarkably, B-GSS appears to have lost the ability to synthesize methionine. Animals including insects generally lack this capability, and the pathway also is absent from the coresident symbiont, *S. muelleri*, from the GSS insect host (Woyke et al. 2010). This is the first reported instance of an EAA pathway lost from all members of an auchenorrhynchan symbiosis, and from a sap-feeding insect symbiosis in general (reviewed by McCutcheon and Moran [2010] and Bennett and Moran [2013]). B-GWSS retains the entire transsulfuration pathway for methionine syntheses (*metABCE*), whereas B-BGSS has lost the initiating genes, *metAB* (fig. 3; Bennett et al. 2014). B-GSS has gone further and purged the remaining two (fig. 3; *metCE*). The loss of *metE* is particularly striking, as it is the terminal catabolic step in methionine biosynthesis. In some other sap-feeding insect symbionts, it is the only gene retained (aphids and mealybugs; Shigenobu et al. 2000; Hansen and Moran 2011; McCutcheon and von Dohlen 2011). It is unclear how GSS acquires methionine. One hypothesis is that the host insect produces it on its own, possibly through microbial horizontal gene transfers (Husnik et al. 2013; Sloan and Moran 2014). An alternative explanation, that methionine is obtained from additional coresident symbionts, although none has been found despite deep sequencing of the insect bacteriomes. Alternatively, GSS may acquire methionine from its food. Xylem feeders have dramatically higher feeding rates than do phloem feeders (up to 1,000× their body weight per day), and they directly assimilate 99% of monomeric amino acids from xylem sap (Raven 1983; Andersen et al. 1989). Methionine is available in xylem at low concentrations, and the amino acid profile of food plants is known to change feeding behavior of other sharpshooter leafhopper species (Brodbeck et al. 1990). Plants also vary in their phloem content of reduced sulfur compounds, including methionine, and grasses, the preferred hosts of GSS, sometimes have relatively high levels (Bourgis et al. 1999). Thus, elimination of this pathway from the B-GSS genome may be linked to a shift in trophic niche of the host insect.

## Accelerated Rates of Molecular Evolution in B-GSS

Given that all strains are of the same origin and age, gene loss is accelerated in B-GSS. The mechanisms driving this pattern are unclear. One hypothesis is that increased mutation rates underlie strain differences between *Baumannia* in the Cicadellini hosts. B-GSS has lost additional repair genes, particularly *mutM* that leads to increased GC to TA mutations (Michaels et al. 1991). Indeed, GC content is much lower in B-GSS than it is B-BGSS (31.9% vs. 39%), and it is slightly lower than in B-GWSS (33.2%). Thus, changes in patterns of molecular evolution may underlie patterns of gene loss (Moran et al. 2009).

**Fig. 4.—:**
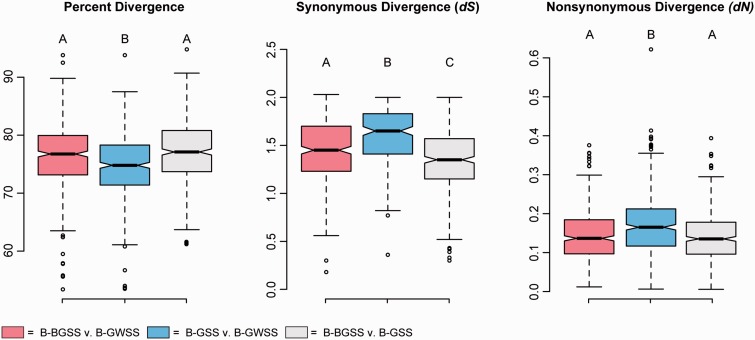
Pair-wise sequence divergence indices for shared genes in each *Buamannia* strain (*n* = 452 genes). Whisker plots show median, quartiles, and maximum and minimum distributions for percent divergence, *dS*, and *dN*. Boxes are color-coded according to each pair-wise comparison (see bottom legend). Statistical significance was estimated using an analysis of variance (ANOVA) and Tukey–Kramer test with a Bonferonni correction. Letters above each plot indicate statistically significant comparisons. Genes that were saturated for *dS* were discarded (see text); no genes were in saturation for *dN*.

Divergence rates are generally high in symbionts, and have been reported to increase in strains that have lost parts of DNA repair mechanisms (Clark et al. 1999; Itoh et al. 2002; Moran et al. 2009; Sloan and Moran 2012, 2013; Gottlieb et al. 2015; Santos-Garcia et al. 2015). To test this between B-GSS and B-BGSS, we estimated pairwise synonymous substitution (*dS*) and nonsynonymous (*dN*) for the core genes shared by all three strains. For each pair-wise genome comparison, *dS* differs significantly (fig. 4; *P* < 0.0001). The lower divergence estimates for B-BGSS versus B-GSS support the closer relationship of their hosts (Takiya et al. 2006). However, for the B-GSS versus B-GWSS comparison, both the *dS* and number of loci at which divergence is saturated (*dS* > 2 for 54% of loci) are significantly higher than they are for comparison between B-BGSS and B-GWSS (21%), or B-BGSS and B-GSS (15%). Similarly, *dN* is significantly higher for B-GSS versus B-GWSS, than for other pair-wise estimates (*P* < 0.0001). Taken together, these results indicate a genome-wide shift in substitution rates in the B-GSS strain. Although some observed nonsynonymous substitutions could be fixed by strong selective sweeps, the higher genome-wide *dN* between B-GSS and B-GWSS, but not B-BGSS and B-GWSS, likely reflects and increased fixation rate of slightly deleterious mutations (Moran 1996; Rispe and Moran 2000; Kuo et al. 2009; Wernegreen 2015). All genes in each pairwise comparison are under purifying selection (*dN*/*dS* < 0.3), indicating that selection is operating to maintain gene function. Thus, it is plausible that acceleration in substitution rates in B-GSS has contributed to increased gene impairment and gene losses.

## Conclusion

Comparative studies of genomes within clades of obligate symbionts are limited, and can elucidate the evolutionary processes that give rise to extreme genome features. Such comparisons are of special interest among the hyperdiverse leafhoppers that contain multiple coresident symbionts. Previous studies demonstrated that interacting genomes coevolve functional complementarity between symbionts and between symbionts and hosts (McCutcheon and Moran 2010, 2012; Bennett et al. 2014). Our results further illuminate the potential mode of symbiont genome degradation by demonstrating that. *Buamannia* genomes deteriorate in a lineage-specific manner. Genomic differences are governed by preceding gene losses and shifting rates of molecular evolution that impact all categories of bacterial cell function (e.g., mutation repair, cell wall synthesis, and nutritional synthesis). Remarkably, B-GSS has even lost the EAA pathway for methionine, which is also absent from the coresident *Sulcia* genome: complete lack of methionine pathway was previously unknown for any auchenorrhynchan symbiotic system. The loss of the ability to synthesize methionine in B-GSS is potentially the extreme outcome of earlier gene losses in the Cicadellini clade and may have impacted host ecology. Although *Baumannia* is ancient, it is relatively young compared with some other insect symbionts (e.g., *Buchnera*, *Carsonella*, *Sulcia*, *Nasuia*, *Zinderia*, etc.). These analyses offer a unique glimpse of how the genomes of established symbionts initially diverge in gene content and then converge upon a tiny genome streamlined for nutrient provisioning.

## Materials and Methods

The yellow bacteriome was dissected out from fifteen individual *D. minerva* adults obtained from greenhouse-reared colonies at University of California at Berkeley. Genomic DNA was extracted, purified, and sequenced using 454 GS FLX following the manufacturer’s protocol. A total of 230,307 reads were assembled with Newbler version 1.1.02.15 into 12,901 contigs of which 29 were of *Baumannia* origin. Potential *Baumannia* contigs were initially binned by GC content and then identified with BLAST. The average contig size was 21,084 bp with an average coverage of 8.7×. Gaps were closed with polymerase chain reaction and Sanger sequencing. RAST and glimmer3 were used for initial gene predictions, and gene identities were determined with Hmmer3 (Aziz et al. 2008; Finn et al. 2011). A total of 68 predicted protein-coding genes were out-of-frame. Since 454 sequencing is known to introduce errors in homopolymer lengths, frameshifts were manually adjusted to be in-frame. Genes disrupted by larger indels were verified with Sanger sequencing. Phylogenetic and molecular analyses were performed in RAxML v7.4.4 and PhyML with custom python scripts as described elsewhere (Yang and Nielsen 2000; Stamatakis 2006; Bennett et al. 2014). Statistical analyses were conducted in JMP v.11.
